# How does vulnerability to COVID-19 vary between communities in England? Developing a Small Area Vulnerability Index (SAVI)

**DOI:** 10.1136/jech-2020-215227

**Published:** 2021-02-04

**Authors:** Konstantinos Daras, Alexandros Alexiou, Tanith C Rose, Iain Buchan, David Taylor-Robinson, Benjamin Barr

**Affiliations:** Department of Public Health, Policy and Systems, University of Liverpool, Liverpool, UK

**Keywords:** PUBLIC HEALTH POLICY, Health inequalities, Disease modeling

## Abstract

**Background:**

During the initial wave of the COVID-19 epidemic in England, several population characteristics were associated with increased risk of mortality—including, age, ethnicity, income deprivation, care home residence and housing conditions. In order to target control measures and plan for future waves of the epidemic, public health agencies need to understand how these vulnerabilities are distributed across and clustered within communities.

**Methods:**

We performed a cross-sectional ecological analysis across 6789 small areas in England. We assessed the association between COVID-19 mortality in each area and five vulnerability measures relating to ethnicity, poverty, prevalence of long-term health conditions, living in care homes and living in overcrowded housing. Estimates from multivariable Poisson regression models were used to derive a Small Area Vulnerability Index.

**Results:**

Four vulnerability measures were independently associated with age-adjusted COVID-19 mortality. Each SD increase in the proportion of the population (1) living in care homes, (2) admitted to hospital in the past 5 years for a long-term health condition, (3) from an ethnic minority background and (4) living in overcrowded housing was associated with a 28%, 19% 8% and 11% increase in age-adjusted COVID-19 mortality rate, respectively.

**Conclusion:**

Vulnerability to COVID-19 was noticeably higher in the North West, West Midlands and North East regions, with high levels of vulnerability clustered in some communities. Our analysis indicates the communities who will be most at risk from a second wave of the pandemic.

## Introduction

Whilst the COVID-19 pandemic has claimed tens of thousands of lives, the impact has not been equal across the UK.^1^ Early in the epidemic, some of the variation between places, was due to how the infections arrived in the UK and then spread across the country. Recent virological findings show that the COVID-19 epidemic arrived in the UK via inbound travel from Spain, France and Italy.^2^ London and more connected urban centres experienced a higher initial seeding of the epidemic. The epidemic then spread along major transport links, reflecting population movement.

Beyond viral transmission, it has also become clear that variation in COVID-19 mortality between places also reflects pre-existing social and health inequalities and patterns of vulnerability. The latest reports from the UK Office for National Statistics (ONS) show that people from Black and other ethnic minority groups are between 1.5 and 3.3 times more likely to die from COVID-19.^3^ In England, the age-standardised COVID-19 mortality rate is twice as high in the most deprived compared with the least deprived areas.^4^ In part, the higher mortality rate in deprived and ethnic minority groups reflects the distribution of pre-existing health conditions that have been shown to increase risk of death in those infected.^5^ These conditions are all more prevalent in these groups because of existing inequalities. The mortality rate in care homes has been particularly high—6.5 times higher for care home residents aged over 85 years old compared with non-care home residents in this age group.^6^ Overcrowded housing has also been linked to increased mortality.^7^ It is likely that the disproportionate impact of the pandemic on these more disadvantaged groups, reflects increased risk of infection through working in high risk, low-skill occupations.^8^ Disadvantaged groups have an additional increased risk of severe consequences among those infected (eg, due to multimorbidity or nutritional status).^1^

The high variability in vulnerability between people and places has important consequences for responses to the pandemic that aim to reduce these inequalities. Recovery strategies and control measures in anticipation of a second wave need to be tailored to differing populations and resources should be allocated proportionate to need. The strategy followed by the UK government so far has tended to be centrally led, drawing criticisms of it bypassing important local expertise such as in local government public health teams.^9^ There are concerns that some approaches—such as reliance on contact tracing apps may not be taken up by the most vulnerable communities, potentially exacerbating inequalities.^10^ Current approaches for allocating resources to local areas have not taken into account specific measures of local population vulnerability, with the UK government moving to a funding allocation on a per capita basis, whereby resources may not be getting to the communities that need them most.^11^

There is an opportunity to learn from the first wave of the pandemic to identify those communities who are most at risk in order to put in place measures to protect those communities from further waves of the epidemic. While there have been a number of small area indices produced to identify vulnerable communities in the UK^12^ these have not been based on empirical analysis of factors that predict mortality from COVID-19. We, therefore, derive a Small Area Vulnerability Index (SAVI) based on the association between a set of vulnerability measures and COVID-19 mortality, while accounting for aspects of the disease spread that are likely to have been specific to the first wave.

## Methods

### Study design and setting

We conducted a cross-sectional ecological analysis across the 6789 Middle Super Output Areas (MSOAs) in England. The MSOAs are the middle geographical level at which census estimates are provided and contain a population between 5000 and 15 000 people (average 8242 people). The Isles of Scilly (E02006781) and City of London (E02006781) MSOAs have been excluded due to missing data in these areas, leaving 6789 MSOAs for analysis.

### Data sources and measures

Our outcome measure is the number of COVID-19 deaths occurring in England between 1 March and 31 May 2020 among residents of each MSOA and registered up to (and including) 6 June 2020 provided by ONS.^4^ The data are based on the entry made by the registrar in the death certificate and COVID-19 reported as underlying cause of death (ICD codes: U07.1 and U07.2).

Since age is a major determinant of COVID-19 mortality, we adjusted for the age profile of each MSOA using national age-specific COVID-19 mortality rates obtained from ONS.^13^ We calculated the expected number of deaths in each MSOA due to age profile alone, by applying the national age-specific COVID-19 mortality to each MSOA’s age-specific mid-year (2018) population estimates. These estimates were used as an offset in the model (see below) in adjusting for age structure differences across MSOAs. To explore the relationship between other vulnerability measures and age-adjusted mortality we calculated the age-standardised mortality ratio for each MSOA as the observed number of deaths divided by the expected number of deaths in each MSOA due to age profile alone.

Our exposure variables were five vulnerability measures derived from the Census 2011 and Hospital Episodes Statistics (HES) at MSOA level. The selection was based on the latest public announcements by the National Health Service (NHS) England^14^ and the WHO^15^ regarding the COVID-19 vulnerable and high-risk groups. First, the proportion of people admitted to hospital for a long-term health condition between 2014 and 2018 was calculated using HES datasets provided by NHS Digital. We calculated the number of people who had at least one admission to hospital and had a diagnosis of cardiovascular disease, chronic respiratory disease, diabetes or chronic kidney disease recorded in their hospital record (see online supplemental appendix A for ICD10 codes included).^15^ We calculated age-standardised proportions of the population admitted for these causes 2014–2018 from each MSOA using the European Standard population.

10.1136/jech-2020-215227.supp1 Supplementary data

Next, the proportion of available care home beds per person was calculated using the Care Quality Commission care directory open data as reported on 1 April 2020. To allocate the number of available beds in each MSOA, we selected the postcodes of locations indicated as Care Homes from the list of active care home providers registered under the Health and Social Care Act.

We then estimated the proportion of ethnic minority groups (Black, Asian and Minority Ethnic, BAME) per MSOA using the 2011 Census data. Next, we calculated the proportion of overcrowded households in each MSOA using the 2011 Census data which classify households in England by occupancy rating based on the number of bedrooms in the household.^16^

Finally, we used the income domain of the 2019 Index of Multiple Deprivation (IMD) for each MSOA as a measure of income deprivation. The income domain of the IMD is a non-overlapping count of the number of people in receipt of welfare benefits due to low income as a proportion of the population.

All five vulnerability measures were standardised using z (SD) scores, by rescaling the original predictors to have a mean of 0 and SD of 1 which assisted us to interpret the model coefficients.

In order to capture aspects of the geographical pattern that were related to the duration of the epidemic in each area, we included the number of days between the date of the first 10 laboratory confirmed COVID-19 cases in each local authority to the 31 May, using data from Public Health England.^17^

### Statistical analysis

First, we explored correlations and distributions of the five vulnerability measures and the log of the standardised mortality ratio in each MSOA. Second, we used a multivariable general estimating equation Poisson regression model to investigate the independent association between each of the five vulnerability measures and COVID-19 mortality, when adjusting for the age profile of each area and controlling for the duration of the epidemic in each area. Nine dummy variables for each Government Office Region were also included in the model to account for broad regional patterns not explained by the vulnerability measures, that were likely to be due to specific transmission dynamics in the first wave. To adjust for the impact of the age profile of the population in each MSOA we included, as an offset in the model, the natural logarithm (log) of the number of deaths in each MSOA that would be expected due to age profile alone assuming the national age-specific rates. Thus, we model the relationship between each of the vulnerability measures and the log of the COVID-19 age-standardised mortality ratio.

A general estimating equation was used to account for the clustering of MSOAs within local authority areas. We visually explored non-linear relationships between vulnerability measures of the outcome and included marginal linear splines to adjust for non-linearity.

Factors related to the distribution of the initial wave (time to first 10 cases and region dummies) were then set to zero. This removes the effect of the duration of the epidemic in each area and regional factors that are likely due to idiosyncratic characteristics of the transmission dynamics in the initial wave—rather than population vulnerabilities per se. The model was then used to predict the number of deaths in each MSOA based on the vulnerability measures and the age profile of each MSOA. This was divided by the number of deaths that would have been expected in each area if it had the average predicted mortality for the country as a whole to give the vulnerability index. This essentially provides a measure for each MSOA that indicates the relative increase in crude COVID-19 mortality risk that results from the level of each of the four vulnerability measures and the age profile for each area.

To further improve the interpretability of the index, we then applied a shrinkage procedure to reduce uncertainty and extreme values in the index by ‘borrowing strength’ from larger or nearby areas.^18^ This method, often used in small area indices recalculates the final index as the weighted combination of the score for each MSOA itself and the mean for the local authority area in which the MSOA resides.^19^ Finally, we mapped the SAVI using the Fisher classification method targeting five group categories where the within variation is minimised, illustrating vulnerability patterns across England and highlighting the MSOAs with the highest levels of vulnerability.

## Results

**Figure 1:**
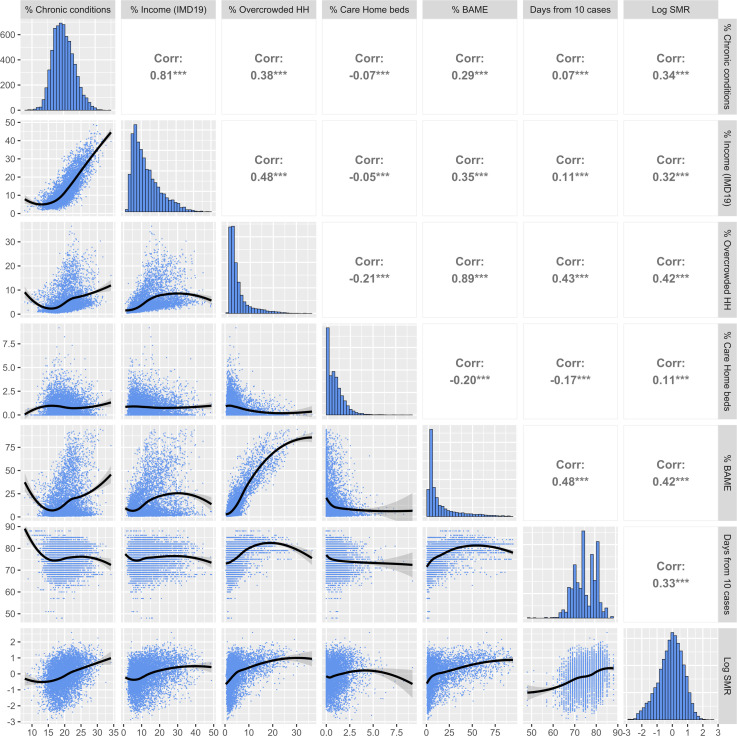
Association between the predictors and the log COVID-19 age-standardised mortality ratio (SMR). BAME, Black, Asian and Minority Ethnic groups; HH, overcrowded households; IMD; Index of Multiple Deprivation.

Figure 1 illustrates the LOESS associations (lower left), the correlations (upper right) and the distributions (diagonal) between the vulnerability measures, the duration of the epidemic in each area and the log of the age-standardised COVID-19 mortality ratio. All vulnerability measures were positively correlated with age-adjusted mortality with the highest correlation found with overcrowded housing and the share of the population from a BAME background. There was some indication that the relationship between age-adjusted mortality and care home beds as a percentage of the population was non-linear with effects reducing at higher values.

The Poisson regression analysis is shown in table 1 (see also online supplemental appendix B). After adjustment for the other variables, income deprivation was no longer associated with age-adjusted COVID-19 mortality and therefore was removed from the model. To account for the non-linear relationship with the care home variable we included a linear marginal spline with a knot at three based on a visual examination of the data. Overall, the model explained 34% of the variation in mortality between MSOAs and Variance Inflation Factors indicated that there were no major issues with multicollinearity^20^ (see online supplemental appendix A: table 2).

**Table 1:** Incidence rate ratios (IRR) showing the relative increase in age-adjusted COVID-19 mortality with each SD increase in relationship between vulnerability indicators and age-adjusted COVID-19 mortality rate for MSOAs in England

	IRR	95% CI	
		Lower limit	Upper limit
Long-term health conditions (%)	1.19***	1.15	1.22
Overcrowding (%)	1.11***	1.06	1.15
Care home beds (up to 3 SD) (%)	1.28***	1.26	1.31
Black, Asian and Minority Ethnic groups (%)	1.08***	1.03	1.13

Poisson model also adjusted for duration of epidemic, region and a linear spline for the care home variable above 3 SD.

*p<0.1, **p<0.05, ***p<0.01.

MSOAs, Middle Super Output Areas.

Each increase by one SD in the proportion of care home beds was associated with a 28% increase in the COVID-19 mortality rate (incidence rate ratio, IRR 1.28, 95% CI 1.26 to 1.31), up to 3 SD above the mean—each additional SD increase after this point was associated with a 12% increase in mortality (IRR 1.12, 95% CI 1.07 to 1.16). Each SD increase in the proportion of the population having been admitted in the past 5 years for a long-term health condition was associated with a 19% increase in the COVID-19 mortality rate (IRR 1.19, 95% CI 1.15 to 1.22).

Each SD increase in the proportion of the population from a BAME background was associated with a 8% increase in the COVID-19 mortality rate (IRR 1.08, 95% CI 1.03 to 1.13), while adjusting for other factors, and each SD increase in the proportion of the population living in overcrowded housing was associated with a 11% increase in the COVID-19 mortality rate (IRR 1.11, 95% CI 1.06 to 1.15), while adjusting for other factors.

**Figure 2:**
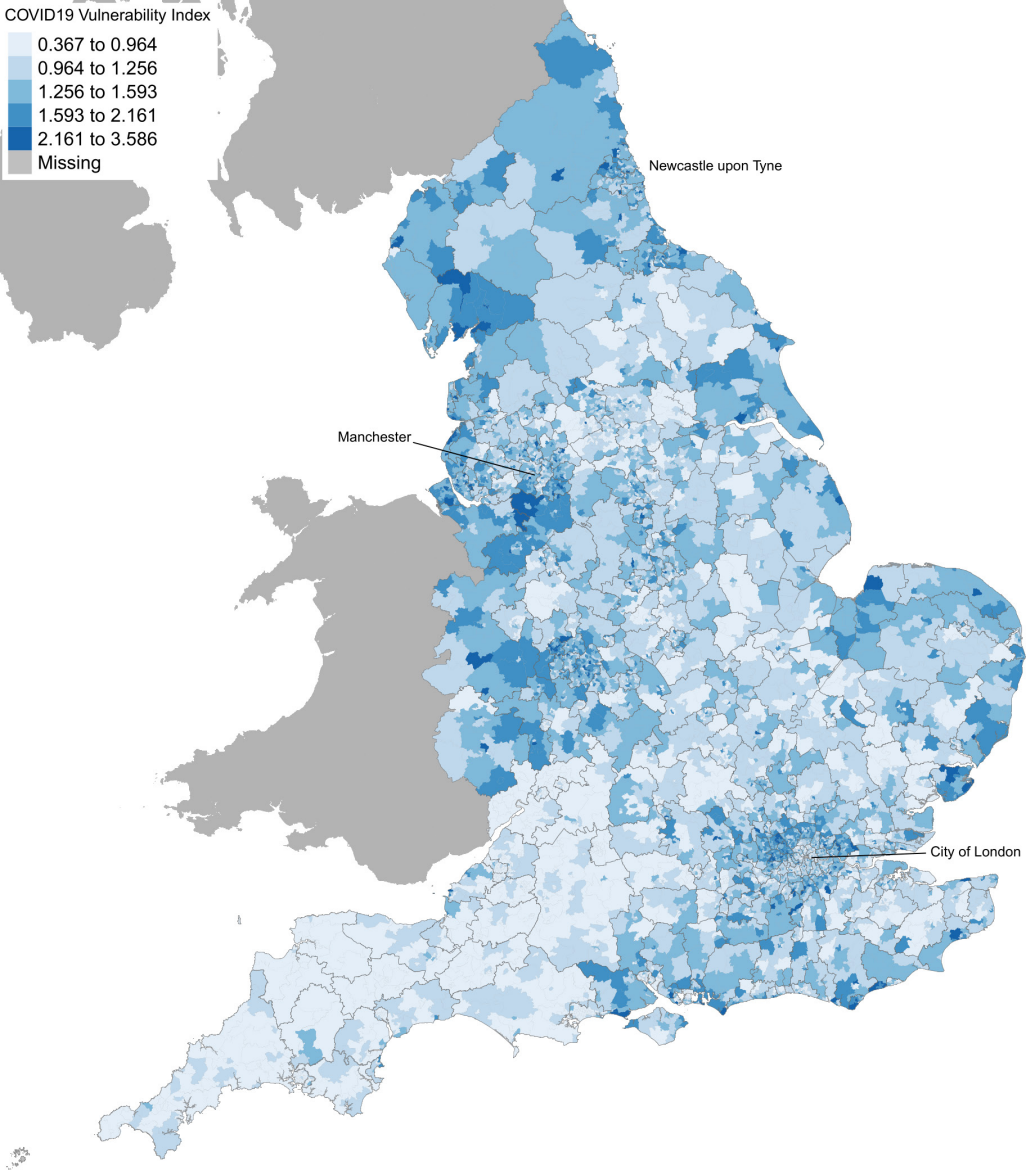
COVID-19 vulnerability index at middle layer super output area in England. Greater COVID-19 vulnerability is shown as darker shade of blue. (MAP copyright statement: contains national statistics data crown copyright and database right 2018. Contains ordnance survey data crown copyright and database right 2018).

We mapped the SAVI (figure 2) using the ‘shrunken’ values for improving the interpretability of the map and explore the geographic distributions of vulnerable communities in England. The most vulnerable to COVID-19 communities are clustered within the North West, West Midlands and North East regions (highlighted with dark blue colour). Neighbourhoods in cities such as London and Birmingham also have clusters of vulnerability. The map shows that the increased risk of COVID-19 mortality in the North is mainly driven by the clustering of underlying vulnerabilities such as overcrowded housing, pre-existing health conditions, age, care home beds per person and ethnicity. Communities in the North and Midlands tend to have highest concentration of these risk factors than in the South. An interactive map including the SAVI and each vulnerability measure is available here: https://pldr.org/2020/06/18/exploring-the-vulnerability-to-covid19-between-communities-in-england/.

## Discussion

Our analysis identifies four important factors independently associated with COVID-19 mortality in addition to age: prevalence of long-term health conditions, living in care homes, living in overcrowded housing and ethnic composition. The association between income deprivation and COVID-19 mortality seen in the univariate analysis was largely explained by increased prevalence of long-term conditions and overcrowded housing which were both also correlated with income deprivation. This suggests that the relationship between COVID-19 mortality and deprivation observed in other studies may be due to inequalities in pre-existing long-term illness and/or the distribution of crowded housing conditions. Similarly, the association between ethnicity and COVID-19 mortality was also reduced when adjusting for long-term condition and overcrowded housing, while it still remained a significant predictor in the multivariable model.

The pattern of vulnerability indicated by mapping the SAVI reveals a distinct geographical pattern resulting from the combination of these four factors population age profiles. The high level of vulnerability in North East and North West is particularly driven by the high levels of underlying health conditions, while having populations with age profiles and shares living in care homes that are not that different to the rest of the population. The West Midlands has a number of compounding vulnerabilities, high proportions of the population from BAME populations, high levels of overcrowded housing as well as relatively higher than average prevalence of underlying health conditions. While London has some vulnerabilities due to high proportions of the population from BAME populations and high levels of overcrowded housing.

There are a number of strengths and limitations to our analysis. We believe this is the first analysis of COVID-19 mortality over a large number of small areas deriving a measure of underlying vulnerability. The use of ecological data such as this indicating vulnerabilities in communities of around 8000 people—provides and analysis at a spatial level that is extremely useful for local policy planning, as this is a realistic size on which to base actions to organise community resilience. Future funding allocation mechanisms can also include such local vulnerability measures to allocate emergency and post-response funding in equitable terms.

Limitations include the lack of data on some potential vulnerabilities. The data we included only explained around 34% of the variation between area, within an epidemic there is likely to be high level of random variation, but also, we may have missed some important determinants of vulnerability. For example, there has been some indication that certain occupations have been more at risk (eg, social care workers, retail workers, taxi drivers)^21^ the distribution of these jobs at a small area level may increase vulnerability. Data on the distribution of employment in these sectors are not available at the MSOA level unfortunately. There remains considerable uncertainty related to the extent to which these and other factors increase vulnerability, however, the SAVI provides an initial starting point for public health agencies to investigate further in combination with other local intelligence in identifying and mitigating risks in vulnerable communities. We have only used aggregate data at the MSOA level rather than individual level data and therefore the associations we observe may not reflect differences in risk at the individual level. Our purpose, however, is not to elucidate causal pathways—rather it is to identify risk factors at the community level.

It is important that responsible agencies learn from the initial wave of the pandemic to prepare for a second wave, which is likely. We cannot know where or when new increases in infection will occur, but these will probably arise out of local reservoirs of infection.^22^ The SAVI indicates those communities that are likely to be most vulnerable when this happens. Without mitigating actions, it is these places that will experience greater excess mortality. These vulnerabilities are clustered in some communities and public health agencies in those places will need to deal with multiple risks. There is an opportunity to step up efforts now to prevent avoidable deaths through increasing funds to the places that need them, supporting actions to reduce vulnerability, while sharing the evidence-based public health measures and intelligence needed to take early action when infections increase.

**What is already known on this subject**

- During the initial wave of the COVID-19 epidemic in England, several population characteristics were associated with increased risk of mortality—including, age, ethnicity, income deprivation, care home residence and housing conditions.
- The high variability in vulnerability between people and places has important consequences for responses to the pandemic that aim to reduce these inequalities.
- Current approaches for allocating resources to local areas have not taken into account specific measures of local population vulnerability.

**What this study adds**

- We identified four important factors independently associated with COVID-19 mortality in addition to age: prevalence of long-term health conditions, living in care homes, living in overcrowded housing and ethnic composition.
- The high level of vulnerability in North East and North West is particularly driven by the high levels of underlying health conditions.
- A second wave of the epidemic is likely to have more severe consequences for those communities identified as highly vulnerable by our index, with disproportionate affects in the North of England and the Midlands.

## Data Availability

Data are available in a public, open access repository. Data are available via the PLDR public access resource: https://pldr.org/dataset/e6kl0.
