# Supplementary material for: How does vulnerability to COVID-19 vary between communities in England? Developing a Small Area Vulnerability Index (SAVI)
Source: J Epidemiol Community Health. 2021 Feb 4;75(8):729–34. doi: 10.1136/jech-2020-215227 (PMC7868127; doi:10.1136/jech-2020-215227)
Supplement: Supplementary data [file jech-2020-215227supp001.pdf]

Supplementary file

Appendix A

Table 1: ICD10 codes selected for creating the proportion of people admitted for a long-term health condition

Long-term condition	ICD10 codes	ICD10 category
Cardiovascular disease	I10-I15	Hypertensive diseases
	I20-I25	Ischaemic heart diseases
	I26-I28	Pulmonary heart disease and diseases of pulmonary circulation
	I30-I52	Other forms of heart disease
	I60-I69	Cerebrovascular diseases
Chronic respiratory disease	J40-J47	Chronic lower respiratory diseases
Diabetes	E10-E14	Diabetes mellitus
Chronic kidney disease	N18	Chronic renal failure

Table 2: Variance inflation factor results for measuring plausible existence of multicollinearity in the model.

Vulnerability measures	GVIF	Df	$GVIF^{(1/(2*Df))}$
Long-term health conditions	1.92	1	1.39
Overcrowding.	5.38	1	2.32
Care home beds	1.14	1	1.07
Black, Asian and Minority Ethnic group	4.97	1	2.23

Appendix B - Model & calculation of the SAVI

The SAVI is calculated from the regression model estimates on the data:

$$log(Y_i) = \beta_0 + \beta_1 Long\text{-}termHealthConditions_i + \beta_2 Overcrowding_i + \beta_3 BAME_i + \beta_4 CareHomeBeds(UpTo3SD)_i + \beta_5 CareHomeBeds(>3SD)_i + \log(ExpectedDeaths\_Age\text{-}adjusted_i)$$

Where Y_i is the number of COVID19 deaths occurring in hospital for MSOA i. *Long-termHealthConditions* is the percent of the population who admitted to hospital in the past 5 years for a long-term health condition (Cardiovascular disease, Chronic respiratory disease, Diabetes, Chronic kidney disease). *Overcrowding* is the percent of the population who living in overcrowded housing. *BAME* is the percent of the population who classify their ethnic group as: Black, Asian, Mixed or Other. *CareHomeBeds(UpTo3SD)* is the percent of the care home beds in each MSOA up to 3SD. *CareHomeBeds(>3SD)* is the percent of the care home beds in

Daras K, *et al. J Epidemiol Community Health* 2021; 75:729–734. doi: 10.1136/jech-2020-215227

each MSOA over 3SD. *ExpectedDeaths_Age-adjusted* is an offset variable which adjusts for the impact of the age profile of the population in each MSOA.

The predicted deaths in MSOA *i* is:

$P_i = exp(0.17 \cdot Long\text{-}termHealthConditions_i + 0.10 \cdot Overcrowding_i + 0.07 \cdot BAME_i + 0.25 \cdot CareHomeBeds(UpTo3SD)_i - 0.14 \cdot CareHomeBeds(>3SD)_i + \log(ExpectedDeaths\_Age\text{-}adjusted_i) - 2.3)$

The final vulnerability index in MSOA *i* is:

$SAVI_i = \quad P_i / Pop_i \times [\sum_{i=1}^{n} P_i] / [\sum_{i=1}^{n} Pop_i]$

Where $SAVI_i$ is the relative increase in crude COVID-19 mortality risk for MSOA *i*. P_i is the predicted deaths in MSOA *i*. Pop_i is the mid-year (2018) population estimates for MSOA *i*.

Daras K, *et al. J Epidemiol Community Health* 2021; 75:729–734. doi: 10.1136/jech-2020-215227
